# The Hospital. Nursing Section

**Published:** 1904-11-05

**Authors:** 


### The Hospital.

### Nursing Section.

Contributions for this Section of "THE HOSPITAL" should be addressed to the EDITOR, "THE HOSPITAL," NURSING SECTION, 28 & 29 Southampton Street, Strand, London, W.C.

No. 945.—VOL. XXXVII. *SATURDAY, NOVEMBER* 5, 1904.

### Notes on News from the Nursing World.

### HOW JAPANESE LADIES HELP THE NURSES.

THE love of economy which, in all departments is a characteristic of the Japanese, is abundantly seen in their nursing arrangements. Their nurses, as we stated when the war with Russia broke out, are only paid £1 a month as salary, and live in the very simplest way, sleeping on two cushions called *futons*, and having so few possessions that they can be contained in a single miniature locker. The food they eat costs so little that it is possible to feed six Japanese nurses for the same sum it would cost to provision a single foreign nurse. Although the nurses are always busy—there are 100 patients to 10 nurses on an average in the Red Cross Hospital—the bandage making is chiefly done for them by an association called the "Ladies' Volunteer Nursing Association." These ladies meet five times a week, princesses, nobles, Japanese and foreign ladies, in a large room at the hospital, often sitting for eight consecutive hours at bandage rolling. They are all obliged to wear the large white overalls and high quaint-looking nurses' caps whilst at work, and must wash and disinfect their hands before passing into the workroom. In less than two months 30,000 bandages have been made by the Association. A few of the members have been selected to help in the wards on certain days when there is an extra pressure of work, and this is considered a high privilege.

### PRINCESS CHRISTIAN AND THE NURSES OF THE SOMERSET HOSPITAL, CAPETOWN.

THE sun shone at Capetown with a brilliance unknown in England the day that Princess Christian paid her long-promised visit to the Somerset Hospital. The exterior was gay with flags, whilst in the interior great palms stood in the entrance hall, and the wards wore a most festive appearance, with masses of flowers, arum lilies, roses, and some very pretty white flowers, not unlike our English "Star of Bethlehem," rejoicing in the odd name of "chinkerry chees." With the flowers were quantities of maidenhair fern, which grows wild on the slopes of Table Mountain. The greater part of the flowers had been gathered by the nurses themselves, and very wet they got in obtaining them. In the nurses' dining-hall were two albums for presentation to Princess Christian and her daughter. The larger album had a cover entirely composed of the famous Cape "silver leaves" all plaited together and lined with white satin, the work of a black girl named Anna Daoma. In the centre of the outside cover were the Royal arms, and this book contained views of the hospital and staff. The smaller album contained snap-shots of some of the wards, taken by one of the sisters. When the royal party arrived, Sister Farish, who had been a member of the nursing staff for the last ten years, presented a bouquet of purple and white iris to Princess Christian, whilst Nurse Thompson, the most recently appointed member, presented one of cream roses to Princess Victoria. The visit of inspection of the wards by the royal party occupied over half an hour. Among the patients most favoured with the Princess's notice was a man who had undergone triple amputation after an accident on the railway, and also an old black man who had been a slave at St. Helena when Napoleon was a prisoner in the island. The Princess commented unfavourably on the men's ward because it had no balcony, and in the nurses' quarters she spoke most emphatically on the urgent need for better accommodation for the staff. Before leaving South Africa the Princess sent a signed photograph of herself as a personal gift to the matron, Miss Child.

### LORD KELVIN'S TIMELY HINT.

IN his address to the prizemen at St. George's Hospital Medical School last Friday, Lord Kelvin observed that every doctor and nurse could administer spiritual consolation to a patient by giving cheerfulness and a kind word or look. Hospital life often tends to make nurses practical to a fault. It is sometimes very difficult to spare time in a ward for sentiment. But a word, a look, taking no time and costing nothing, will frequently, as Lord Kelvin also said, be waited for from a sick bed throughout a weary day. We know that this is so; and that if that expected word or look be not forthcoming the sufferer's day is a blank, since in illness everything is viewed disproportionately. The subtle sympathy of a real nurse makes her feel that no small part of the art of healing consists in trying to instil hope and cheer into the helpless ones at her mercy.

### THE AUSTRALIAN ARMY NURSING SERVICE.

IN a series of lectures which have been recently given at the Victoria Barracks to the nurses of the Victorian Army Nursing Reserve, Colonel Ryan stated that he considered in actual work the *personnel* of the Australian Army Nursing Service as now laid down would be found far too small. To a "general hospital" of 520 beds under the administration and command of a senior officer of the medical service there are assigned one matron, eight nursing sisters, and two female servants, as well as orderlies for general duty, nursing, and clerical duties. This staff, Colonel Ryan thinks, would be altogether inadequate to deal with an influx of severe gunshot wounds or enteric, and would need to be considerably reinforced. But the details of the service have been occupying the attention of the Director-General for some time, and a more complete scheme is likely to be put forward at an early date. The somewhat complex duties of a matron at present render her unaccountable for the general nursing arrangements of the hospital, supervision of the nursing training of the non-commissioned officers and men of the Army Medical Corps, such share of the nursing as may be ordered by the lady superintendent—or matron-in-chief—and responsibility for the condition of the bedding and linen. She has also the keeping of all books and accounts connected with the nursing staff, including their mess. The nursing sisters are responsible for the cleanliness of the wards and the patients, and for the nurse training of the orderlies.

### THE BISHOP AT CRUMPSALL INFIRMARY.

A few days ago the Bishop of Manchester paid an official visit to the Crumpsall Infirmary. He was accompanied by Mrs. Knox and by a large party interested in nursing. They were received at the entrance by Miss Girdlestone, the infirmary matron, and her assistant matrons, and by the resident medical staff. The bishop then visited the wards and spoke very kindly to many of the patients. Everybody had done their utmost in the way of preparation, and the well-polished floors, the beds with their pretty scarlet and white quilts, and the tables gay with flowers and plants, were much admired by the visitors. After tea the bishop gave an address to the nursing staff in their sitting-room, in the course of which he spoke of the pleasure it had given him to see the bright cheerful-looking wards and to know that the sick poor of his cathedral city were cared for and helped in their necessity by every possible means. The care of the sick was the duty of the whole Christian body, but the actual work could not be done by all, and in the name of all he thanked the nurses for doing it. The bishop then dwelt on the need of care, lest in the great interest of the technical part of their work, nurses should forget the human side of it. Lectures and certificates were necessary, no doubt, but no lectures could give either a kind heart or the gentleness and sympathy essential to the highest type of nurse. Dr. Reynolds, in seconding a vote of thanks to the bishop, said that the day was the jubilee of Miss Nightingale's entrance on her work in the Crimea, and that, although nursing had now become a recognised profession, the same spirit that inspired Miss Nightingale was still found among nurses at the present day.

### THE PENSION FUND AND NORWICH NURSES.

By the courtesy of the Board of Management of the Norfolk and Norwich Hospital, Mr. Louis H. M. Dick gave an address to the nurses on Wednesday last week on the aims and objects of the Royal National Pension Fund. The chair was taken by Dr. Barton, senior physician of the hospital. There was an attendance of over 100, including Dr. Thomson, medical superintendent of the Norfolk County Asylum, Thorpe; Miss Cann, matron of the hospital; Mr. Frank Hazell, the house governor; Miss Oxley, matron of the Bethel Hospital; Miss Sunderland, matron of the Jenny Lind Hospital, and the matron of the Thorpe Asylum. After the meeting the visitors were entertained at tea by Miss Cann, to whose energy the success of the meeting was largely due. The hall of the hospital presented a charming and attractive appearance owing to the trouble which had been taken in the decoration. It will be learnt with general interest that upwards of a thousand policies have already been issued during 1904 by the Pension Fund, a larger number in ten months than during any completed year.

### THE RECOGNITION OF THE CENTRAL MIDWIVES BOARD.

It appears that a good many institutions, which the Central Midwives Board has so far been unable to recognise, do not, judging by communications which they have subsequently addressed to the Society, know how to account for the refusal. In some cases the difficulty seems to arise from a want of comprehension of the Board's requirements, which are very definite, and a somewhat confused method of filling in the application forms. We may therefore point out that the Board requires a certain number of cases to be conducted personally by each pupil. In order to secure this it is obvious that each institution must limit its pupils to the number of cases that can be provided. Yet in many applications there is an entire want of proportion between the number of available cases and the number of pupils claimed to be trained. Institutions wishing for recognition should take note of this fact, and see that they do no needlessly put themselves out of count by what is apparently a misapprehension of the conditions to be observed.

### THE LOCAL GOVERNMENT BOARD AND THE AGE QUESTION.

It is announced that the Local Government Board have not only refused to sanction the appointment of a young woman aged 19 to the nursing staff of the Poplar and Stepney Sick Asylum, but have also stated that they are not prepared to approve that of "any persons under 21" as nurses. This official intimation that the department does not consider anyone under 21 competent to undertake the duty of nursing the sick poor will not be acceptable in certain quarters. But whether in the interests of patients or that of nurses generally, it will be welcomed, and cannot fail to have a good influence in the direction of raising the standard of workhouse nursing.

### THE COUNTY ASSOCIATION FOR DEVONSHIRE.

The meeting convened by Lady Ebrington at Exeter for the purpose of forming a county nursing association for Devon was well attended, and many influential county people who could not be present wrote expressing sympathy with the movement. Viscount Ebrington, who presided, mentioned that in the county of 500 parishes there were fewer than 50 village nurses—altogether an inadequate number for so large a county, and still more inadequate in view of the passing of the Midwives Act Miss Amy Hughes, superintendent of the County Nursing Association in affiliation with Queen Victoria's Jubilee Institute, gave an interesting address, and announced that if the Association was formed upon certain lines which the other county associations had adopted, the Queen's Institute would give a grant to the Association of £100. The Institute also offered to pay £50 a year towards a county superintendent, provided the report of her work was satisfactory. Mr. S. Sanders Stephens, sheriff of Devon, moved a resolution in favour of forming an association, which was seconded by Lady Portsmouth, supported by Sir J. Kennaway, M.P., and unanimously agreed to. The annual sum required is £500, and as annual subscriptions promised before the meeting amounted to £183, while donations exceeded £200, the strong executive committee which was chosen should experience little difficulty in securing sufficient funds, especially as in the adjoining county of Somerset, which is smaller than Devon, £530 a year is raised for a similar purpose in subscriptions.

### THE NURSES' UNION.

On Tuesday last week Miss Marion Dashwood gave an "At Home," with which was combined a missionary sale of work, at her residence, 5 Cambridge Gate, Regent's Park. All the work for the sale was done by nurses who are members of the Union. The Rev. E. G. Hodge, rector of Holy Trinity, Marylebone, opened the sale at three o'clock, and after tea Sister Lucy, late of Guy's Hospital, gave a graphic account of her experiences in nursing the Boer prisoners in Ceylon. Dr. Jessie Gray, of Benares, next gave an interesting description of the work of doctors and nurses in the Victoria Hospital, and also in the Memorial Hospital, Lucknow, where the Nurses' Union support native nurses. These nurses, she said, were always ready to volunteer for dangerous and difficult work if needed.

### THE TRAINING OF THE MATRON OF LEWISHAM INFIRMARY.

At the last meeting of the Lewisham Guardians a letter was read from Dr. Rose Bradford stating that he had examined Miss Strick, matron of the infirmary, orally, in a manner similar to that in use for the examination of nurses of the Lewisham Infirmary. In his opinion, she possessed a sound, practical knowledge of her work; and her answers showed that her training had been complete and thorough. He added that upon that report a certificate had been submitted to him, which he had signed. Miss Strick herself submitted certificates of nursing, one for first year's training, another for two years, and a third for three years, and expressed the hope that these would be found satisfactory. We have no doubt that with this documentary evidence of her personal qualifications for the post of matron before them, the Local Government Board will concur in her appointment at Lewisham.

### RURAL NURSES IN YORKSHIRE.

The Marchioness of Zetland is appealing for funds for the North Riding Rural Nursing Association. This organisation was formed in 1891 with one nurse, and there are now on the staff, Lady Zetland states, 27 nurses, five probationers, and one assistant probationer. As to the training, the persons selected by the Committee of the Association are three months at Northallerton Cottage Hospital, and if found suitable at the end of that time are engaged for five years, the first year being one of probation, during which special training at Sheffield, Middlesbrough, or Darlington, is given for hospital work, and at Glasgow for maternity work and district nursing. All this is very well as far as it goes, but it seems a pity that the Association is not affiliated to and working in connection with Queen Victoria's Jubilee Institute. At present the subscriptions, donations, and fees are insufficient to meet the outlay, hence Lady Zetland's appeal. We are satisfied that it is sound policy to aim at the reduction of organisations formed on an independent basis, and that the more they are increased or extended, the more difficult it becomes for the public to understand the status of the nurses.

### ANNUAL MEETING OF THE NURSES' HOSTEL COMPANY.

The seventh annual general meeting of shareholders of the Nurses' Hostel Company was held last week. The report and balance-sheet were adopted, and the dividend of 4 per cent. recommended by the directors adopted. The shareholders also approved the capital redemption scheme proposed by the directors, so that there will henceforth be a sinking fund which will maintain the shares at their full value when the time approaches for the lease of the two houses to fall in to the ground landlord. An interesting item in the report is that, while the number of visitors in the year has increased by 33, the total of visits paid is less by 173 than it was in the previous twelve months. But that the average length of stay of each nurse in the hostel has been longer is proved by the fact that, whereas during 1902–3 the average number of nurses sleeping in the buildings each night was 72, it rose in the year just ended to 78. All the unfurnished rooms in the south block are let, and the report states that any that fell vacant in the course of the year were taken at once by nurses who had been waiting to secure them.

### A CHOIR OF NURSES.

Following the annual service for the staff at St. Mary's Hospital, Paddington, on Tuesday last week, at which the Dean of Westminster preached the sermon, a harvest thanksgiving service was held on Sunday morning. The choir was composed entirely of nurses, who had been carefully trained by the Home Sister. The anthem, "The Lord setteth fast the mountains," was well rendered, and the patients joined heartily in the singing of the thanksgiving hymn.

### DEATH FROM MORPHINE SULPHATE.

The death of a nurse at Walsall a few days ago was followed by an inquest, at which it transpired that she had been for six months attending an elderly lady. Her duties were said to be light, and she was described as being of a bright and cheerful disposition. The night before her death she was laughing and joking, and the discovery in the morning that she was in a comatose condition, the symptoms pointing to poisoning by morphia, caused painful surprise. The usual remedies were applied, but the nurse died during the day. In a box belonging to her an empty tube, labelled "Poison—morphine sulphate," was found, and the doctor in attendance stated that the nurse had dissolved two or three of the pellets, as a cup containing the drains was in the bedroom. The jury properly returned a verdict of "Death from misadventure," but the event is a warning to nurses not to take drugs for any purpose whatever unless they are prescribed by a medical man.

### The Nursing Outlook.

"From magnanimity, all fear above;
From nobler recompense, above applause,
Which owes to man's short outlook all its charm."

### WINTER ABROAD.

A nurse has to go where her work is; hence the private nurse has often to follow in the wake of the annual rush for the Riviera and Egypt of the rich who seek for health and for escape from London fogs. Most English people who can afford the time and money now winter abroad, for yearly our atmosphere becomes more laden with smoke and dust, and our breathing spaces smaller. Those two great life-givers and disinfectants, light and fresh air, are conspicuous by their absence during a London winter, and it is small wonder that those who want pleasure and those who want health should alike seek some brighter climate. But many take their ailments with them, and so there have grown up at Nice and Florence, at Rome and Palermo, at Algiers and Cairo, certain centres of English nurses—not always institutions, but sometimes homes in which a few nurses live together. This is a nice change for the private nurse; she also escapes the fogs and enjoys the sunshine, she sees news sides of life, and widens her experience and increases her knowledge.

The main difficulty is taking the first step, for the nurse who would make a winter abroad successful must have a little capital and an acquaintance with at least one foreign language. It is generally the language that is the stumbling block. The French of the schoolroom is useless stuff as a rule; it never included phrases which taught us how to take return tickets or buy postcards; it was mostly concerned with the "horses of my aunt," or "the penknife of my cousin." But though it is difficult for most of us to learn any foreign language thoroughly through the medium of grammars and exercises, it is possible to most of us to pick up the useful everyday phrases of a country if only we will go and live amongst the people for a brief period. A nurse who has the courage to go alone on a walking tour in Normandy or Brittany, armed only with a phrase-book and a dictionary, should at the end of ten days have learnt thoroughly all the French that is necessary for travel. In the same way, a fortnight in one of the little Italian towns, where no one speaks English, will teach you more useful Italian than several years of study. For the woman nursing in a foreign country needs not only to know the language, but the manners and habits, the limitations, as it were, of her surroundings. Both French and Italian cookery are eminently adapted for invalids; but if you try to order exclusively English dishes for your patient, because, for instance, you have never heard of *risotto* or *panna*, you will get but wretched results, and unless you understand the friendly relations of mistress and maid in continental countries, you will give deadly affront to the servants, and your wishes will not receive due consideration.

The nurse who seeks success in many lands must try and drop all insular prejudices and get into harmony with the inhabitants of the country she is in; it is not only that she will be happier in herself if she sees "good in everything," and that her work will be easier, but she will also create an atmosphere of peace and rest for her patient which is one of the main elements of cure. A most excellent English nurse may so upset an Algerian household if she is not tactful, that all her skill will be outweighed by the discomfort she will cause and the trouble she will give. No nurse should seek to winter abroad who is so hide-bound that she grumbles if she has coffee instead of tea in the morning, and if bacon is an impossible dish. Such narrow people should remain within the narrow wards, and most certainly should not adventure out into other lands.

And then those who go abroad must place no limits to their duties; whatsoever concerns their patient must be their concern, and they must be ready to do whatsoever is needed with all their might. Here is a perfectly true picture of what happened last spring to a Canadian nurse. She was sent to an up-country typhoid case, a journey of some hundreds of miles; at the end of the railway she got on a stage, but the driver told her he did not think they could get over the pass as snow was falling on the summit. She knew the case was serious, so telephoned on to the next changing place for an extra horse to be in readiness, and with difficulty the journey was accomplished, and she was landed at a little log cabin after three days' travelling. She found the patient—the only woman in the little settlement—very ill, and in a terrible state of dirt and neglect, with a huge bedsore. It rained and snowed for several days, and not the least difficulty was to get the linen dried after it was washed. There was only a small cooking stove in the outer room. The doctor in charge was, unfortunately, addicted to morphia; he was constantly changing the treatment and worrying the nurse, and so far lost to self-respect as to beg from the nurse her syringe and needles, his own being clogged and dirty from constant careless use. The nurse could only watch and work and do her best, and it so happened it was all in vain, for the patient died. The nurse wrapped her in her shroud, took the only child back with her to more civilised regions, and left the poor husband to live on alone in his little log-hut. She also left behind her a memory of what a good woman can do in hours of danger and distress. The one great thing to impress is, that only those who have courage and tact should set their feet to the difficult paths. The timid nurse should stay at home.

### Lectures Upon the Nursing of Mental Diseases.

By ROBERT JONES, M.D.Lond., B.S., F.R.C.S.Eng., M.R.C.P.Lond., Resident Physician and Superintendent of the London County Asylum, Claybury.

### LECTURE XXI.

We have dealt with the nursing of insane persons for diseases of the chest and referred to those affecting the heart and arteries, the lungs and their covering, the pleura. We shall in the present lecture deal with disorders of the abdominal organs, and firstly, those of the digestive system, which are by no means easy to discover in persons suffering from mental disease, as pain—which is the most definite and general symptom—may be and often is entirely absent, or, if present, rarely rises into consciousness. *Dyspepsia* or indigestion is not common among inmates of asylums owing to the plainness of the food, the regularity of the service, and its limited quantity, determined by a well-thought-out scheme; so that the various aches, pains, and discomfort about the chest, pit of the stomach, shoulder-blades, and sides, indicating indigestion, are uncommon among these persons. Flatulence and eructations, fulness, acidity, headache, nausea, sickness, and vomiting, which are the usual symptoms of dyspepsia, rarely indicate this condition among insane people. When present they may be symptoms of much more serious conditions, such as cancer or some other grave disease of the stomach or the intestines. So often is this true, that whenever an insane person suffers from vomiting, internal strangulation may well be suspected and should be looked for; more especially must the nurse be satisfied that there is no hernia—inguinal, femoral, or umbilical—which has possibly become strangulated at one or other of the natural openings in the abdominal wall to account for the symptoms. I have even known a strangulated hernia overlooked by a medical man, and the urgent symptoms treated as from severe gastritis. Always therefore ascertain the presence or absence of a rupture or hernia at one of the usual seats, either at the navel or in the groin, and any doubt upon the matter should be immediately reported to the medical officer.

The first consideration in regard to the digestive system in the order of sequence is (A) to notice whether the food is properly masticated and (B) whether it is swallowed without difficulty. Many insane persons such as those who are suffering from the dementia of general paralysis—although they have previously to their insanity been persons of essential refinement and cultured manners—become reduced and degraded through their infirmity into creatures of purely reflex action, only their animal instincts remaining. These eat with avidity, and even collect the refuse food left on others' plates, becoming mere scavengers. They "bolt" their meals and may, yea sometimes do, run great risks of choking, to which we shall refer later. It is the duty of the mental nurse to be especially watchful during the serving and partaking of meals, so that bones, gristle, or masses of unmasticated food are not swallowed and do not thus obstruct the upper air-passages or the gullet, and to call immediate attention to any complaint or apparent difficulty in masticating or swallowing. For such patients, specially prepared food carefully minced and with appropriate vegetables, is always ordered. When vomiting occurs the matter ejected should be kept for the inspection of the medical officer, as the appearance of (*a*) undigested food with the peculiar sour acid odour due to the action of the gastric juice; (*b*) foreign matter, such as something swallowed which is not food, leaves, grass, bits of wood, dirt, or pebbles, which cause mechanical irritation; or (*c*) blood, and (*d*) "fœcal vomit" may be an indication of the cause of the sickness. Vomiting in the early mornings is not an uncommon symptom, in both sexes, of alcoholic mental affections, and it is a normal symptom of early pregnancy. It may also indicate serious brain disease, such as pressure from tumour or abscess, and may it accompany kidney or uterine affections. The vomiting of undigested food in large quantities may probably point to dyspepsia as the result of cancer of the stomach, which becomes dilated or distended, the food undergoing decomposition and fermentation. Persistent vomiting of foul smelling matter or "fœcal vomit" indicates intestinal obstruction, and the vomiting of blood is a common symptom of gastric ulcer, or congestion from disease of the liver, as may occur in heart affections and in cases of alcoholic insanity with cirrhosis. The appearance of the blood may be dark brown, or it may resemble coffee-grounds, owing to the action upon it of the gastric juice, and there may be an admixture of food with the blood. Vomiting of blood may occur as an isolated attack, or such an attack may be repeated, and the hæmorrhage may so endanger life.

*Ulcer of the stomach* occurs mostly in young women who are anæmic and suffer from chronic indigestion. There is often tenderness on pressure, even if there is no actual pain, and it is increased on pressure in these cases. There is discomfort after food, followed by vomiting, which may relieve the pain. In some cases perforation of the stomach takes place at the seat of the ulcer, which thus eats its way through the peritoneum and permits of the contents of the stomach escaping into the peritoneal cavity, causing sudden death from shock, or a less rapid death—in one or two days —from peritonitis. *Constipation* is a common condition among insane persons, especially females. It is a frequent cause of diarrhœa, and it may be the cause of intestinal "*colic*." The patient may bend double with the pain, which is thus relieved by pressure and warmth. Colic may also be a symptom of inflammation or obstruction of the bowels.

Diarrhœa is a frequent ailment among the insane, mainly because they are unable, owing to their mental infirmity, to exercise care in the selection of food, often eating what is unsuitable or indigestible, and thus giving rise to irritation of the intestinal tract, partly also because it may be communicated to them by others. It may be caused by cold, or by sudden variations in the temperature of the air. It may occur in the form of *English cholera*, or *summer diarrhœa*, as an epidemic, with pain, cramp, profuse discharge, cold extremities, and feeble pulse, and giving rise to fatal exhaustion and collapse. In like manner it may be endemic among a people, as is exemplified in the colitis or dysentery seen among those who live in large institutions, asylums, or workhouses, more especially when, as is often the case, there is over-crowding.

In some of these institutions this form of dysenteric diarrhœa has become a perfect scourge, and the symptoms which characterise it, viz. (*a*) loose motions, with (*b*) blood and *mucus*, or slime; (*c*) straining or *tenesmus*, together with (*d*) general weakness, are an indication for isolating the patient from others. When the stools are black, the blackness, called *melæna*, may be caused by blood which has been acted upon by the gastric juice; in such cases the hæmorrhage is probably high up and may be caused by a gastric ulcer. When the blood is red, it is probably from the large intestine low down, or it may be from piles or hæmorrhoids within the rectum. The nurse is always instructed and requested to wash her hands after changing or handling the clothes of such patients, to convey foul linen to proper receptacles, to keep special feeding utensils for these patients, to cleanse and disinfect the nozzle of enema syringes after use, and to observe all other special regulations that may be issued for the prevention of the spreading of this disease, such as identifying them by special case-cards, examining the *dejecta* once a week or oftener, and noticing whether any tendency occurs to loose motions, and that all patients and others associated with them should carefully wash before each meal. It is important that the stools or motions of all patients suffering from diarrhœa be kept for examination by the medical officer, and the nurse should herself notice the amount, consistence, colour, odour, and general constituents in regard to foreign bodies or substances such as undigested food, seeds, gall-stones or fruit-stones, skins, intestinal worms or membranes which may be passed by the patient.

*(To be continued.)*

### Nursing in the Russian-Dutch Field Hospital at Taolaitschao.

THE following is a translation from the letter of a nurse published in the *Petersburger Zeitung*, which was written on August 29th.

"On the night of the 24th, we, as well as all other field hospitals, had to leave Mukden and retreat north on account of the approach of the enemy. After a journey of two days we reached Taolaitschao, where we were to fix up a hospital and spend the winter months. But since the recent news from the front, we think such a long stay unlikely. All the same, we hope to be able to work here quietly and diligently for at least two months. A long stone building—the barracks of the frontier guard—and several smaller houses as quarters of the staff, have been kindly placed at our disposal. Everything is near to the railway line. We have also a part of the station—the waiting-room—as depôt for our stores. In the station itself we are to arrange a temporary 'house of succour,' so that in case hospital-trains pass after a battle without attendants and necessaries, they may be provided with food and medical help. After the battle of Liao-yang the wounded had often to be put into goods-trains and be despatched to Chanbri without any provision for their comfort; and when they arrived they sometimes had to wait several days at the station before room was made for them in the hospital. Work will not fail us here; all field hospitals along the line are overfilled, and the diligent Dutch are not left long without something to do.

### Work at Mukden.

"The last days in Mukden—the 21st to the 23rd—were very hard ones, difficult for us nurses, not only from a physical, but also from a moral point of view. Our hospital was quite full when, on Aug. 28th, we were told to turn out all patients to make room for the expected wounded. The same afternoon we handed over our patients, all except a few dangerously ill, to a hospital train. One sister and a member of the sanitary corps were sent with them. The same evening a doctor, a student, and three sisters departed to Liao-yang with dressings for the wounded left there. So only a small portion of the staff was left at Mukden, and it had to act promptly to master the work which shortly fell to its share.

### A Train Full of Wounded.

"At five o'clock on the morning of Aug. 21, an urgent appeal was received for medical help for a train full of wounded, arriving from Liao-yang; a doctor, two students and one nurse hurried to the station, and dressed over 100 patients in a few hours. In the meantime various ambulances laden with wounded arrived at the hospital, and were quickly taken in and dressed. This was repeated several times until nearly all beds were full. They were all severely injured, the cases being either of thoracic, abdominal, or head gunshot wounds. The greater number were already marked down as the prey of Death. They lay writhing with pain on the stretchers, their clothes, such as they were, dirty and torn, many half naked.

### Terrible Scenes.

"One could have cried out aloud at the harrowing spectacle. Until it was dark we dressed wounds unceasingly. We were so weary that at the last we worked like machines, and questions even remained unanswered, the tired brain could not grasp the drift of them sufficiently to reply. One person had enough to do to administer morphia to allay pain. The injuries were terrible. For instance, one man was wounded in the head; he was unconscious, and when the bandage was taken off, it was found that the skull was open and the brain lay bare. Another had been shot through the spine; he lay paralysed in both legs, and he had one arm shot through as well. And so on from bed to bed, one dangerous case after another.

### Dying Men in each Division.

"When evening came there lay dying men in each division; we four nurses went from one to another, comforting and soothing. Many a tear fell on to the sunbrowned face of a poor soldier struggling with death. Many a young life fought with death, and could and would not grasp that he must die—die when he had a young wife and little children at home. Many a soldier in the pride of health and strength, hopelessly wounded, asked the despairing question. Many wandered in their minds, called us by the names of their wives and children; some gave us their savings, with the request that we would send them to their wives and write to them. And all around men groaned and wailed, and through the evening quiet the call for 'Sister' rang as soon as we entered the tent. Only 'Sister' could help, only 'Sister' could lay them more comfortably. We received many a grateful look of thanks and pressure of the hand. But what the sisters felt in their secret hearts, and how they suffered morally, that God only can know. He alone saw how, from time to time, they hurried from the tent to hide their tears, and to wring their hands as they struggled to regain their self-control."

### Death in Our Ranks.

WE regret to hear of the death of Miss Alice H. Banham, formerly matron of the Sanatorium at the Surrey Public School at Cranleigh. She was trained at Addenbrooke's Hospital, Cambridge, and then became sister at Ayr Hospital. She returned after some time to England, and was assistant matron at the Royal Berkshire Hospital for four years. In 1894 she was appointed to the matronship at Cranleigh, which she held for eight years, retiring on account of ill-health.

### TRAVEL NOTES AND QUERIES.

By our Travel Correspondent.

Homes for Nurses in Paris and Rome (Tourista).—I do not think such a place exists for your purpose. Your two friends, as I understand, are not wanting accommodation as professional women, but simply as visitors to these cities, therefore they would have no claim on such institutions and must be content to live as other women of small means do when travelling. There are, I believe, Homes in both places for nurses, but only in their professional capacity. If I can help you to ordinary cheap accommodation I shall be pleased to do what I can.

### The Prevention of Scarlet Fever Infection.

### EXAMINATION QUESTIONS FOR NURSES.

The question was as follows:—If called upon to nurse a case of scarlet fever, in a house where there were other inmates, what steps should you take for the prevention of the spread of infection?

### First Prize.

"To prevent the spread of infection in Nursing a case of Scarlet Fever." Choose a room in the top, or out-of-the-way part of the house, so as to be able to shut off communication as far as possible with the rest of the house.

In preparing the room only admit articles of furniture that can be subsequently disinfected easily. It is as well that carpets, curtains, and surplus clothes should also be removed.

Discourage as far as possible all visits to the patient by other inmates of the house, and where such restrictions cannot be strictly enforced, children should at all events never be allowed in the room. Hang before the door a sheet saturated with a disinfectant solution, which must be kept constantly moist, the object being to catch the dust from the sick-room, and prevent it being blown about the house.

During the course of the case the following rules should be observed.

All discharges from the nose and throat should be received on pieces of lint or rag and immediately burnt

Remnants of food are best treated in the same way. They should never be taken from the sick-room and consumed by other people.

Cleanse all vessels used by the patient, firstly in a solution of carbolic acid, and afterwards by boiling water. All soiled portions of linen, towels, etc., should be placed in a large pan and covered with a carbolic solution 1 in 20, and then squeezed out and placed in a large tub containing carbolic solution 1 in 16 until such time as they can be boiled.

Have in the room (if possible) a tin box the size of a travelling-trunk, and into this place any infected article, such as blankets, etc., so that when removed to the steam-disinfector the box can be taken away and another substituted in its place. All useless and worn-out articles should be burned. Enforce the isolation until the peeling has finished and until any discharge from the nose and ears have cleared up.

During the period of desquamation (if the physician permits) oil the skin daily after bathing to prevent the small scraps of skin from becoming detached and possibly blown about the house.

Flush all drains, sinks, and water-closets with a strong carbolic solution daily.

During his attendance on the case the nurse should be very careful himself not to carry about infection. His infected clothes must be kept separate from his others.

At the conclusion of the case the patient should have a hot bath in his room, putting on clean clothes in an adjoining room. The room and its contents should then be thoroughly disinfected, and any articles of furniture which may have come in contact with the patient during his illness should be included in this fumigation. The room then requires the attention of the sanitary officials. Ebor.

### Second Prize.

I should, if possible, secure a room at the top of the house, remove from thence all unnecessary furniture, together with window-blinds, carpets, bed draperies, etc. Then get a sheet, wring it out of a disinfectant (carbolic 1 in 20) or any other at hand, and hang it outside the patient's bedroom door, taking care it was large enough to cover all crevices; this sheet I should keep damp with the disinfectant until all means of infection have ceased (*i.e.* after desquamation). I should secure all utensils necessary for use in the sick-room; such as cups, spoons, etc, and a "washing-up" bowl, in which I should wash cups and other vessels used by the patient, being careful that nothing used by the latter found its way to the kitchen or other rooms.

All food required I would have deposited at some distance from the door of room, to where, after first disinfecting my hands, I would take one (or more if necessary) of the patient's utensils and transfer the food from the one brought up to the one I had, leaving the former there to be taken back again.

For the removal of excreta I would have a pail containing a disinfectant covered all over with an old towel or piece of cloth, also wet with disinfectant, into which I would put all excreta, emptying it myself if the W.C. be near the room, if not, putting it outside the door, and making arrangements beforehand with a maid or other inmate to empty it periodically.

During desquamation I should oil the patient's body every day after giving the disinfecting bath, to prevent the particles of skin from being transmitted by the air to other parts of the house. I should thoroughly disinfect all bed linen, body linen, and everything coming in contact with the patient and myself before sending to the laundry. I should myself have no intercourse whatever with the other inmates of the house, giving my orders for food, etc., from a distance, or on a slip of paper previously disinfected. I would have my food brought in the same way as the patient's, and eat it in my own room.

I would change all uniform used in the patient's room before going out, and pass through the house as quickly as possible. I should also adopt the plan of having a sheet well sprinkled with carbolic which I would wrap round me when passing to my room, thereby avoiding the risk of disseminating microbes from my uniform. I should dust everything in the patient's room, including the floor, two or three times a day with a carbolised duster, burning the sweepings of the room, or if there is not a fire in the room, saturating them with a disinfectant before disposing of them. Patricia.

### Large Number of Papers Sent In.

So great has been the competition this month that my work has been difficult, very many answers differing but little in order of merit. Taken as a whole, the work is very good, but it would have been more satisfactory if intelligent reasons had been given for the almost universal statement that a room at the top of the house is the best for a case of infectious disease. There are several reasons, but there is one of more importance than all others. Only two nurses have mentioned this, though probably a large proportion of the competing nurses are aware of it. In the spring a question will be given to draw forth whatever knowledge nurses possess on the matter.

"Ebor" wins the First Prize, his paper being the best available. He shows practical knowledge, and his idea of the tin box is good. He is evidently fully aware of the necessity of extreme care being taken by the nurse not to convey infection himself when on necessary errands to others outside the sick room.

"Patricia" is successful as the Second Prize winner. She has sound views as to fetching food and transferring it to other vessels, so as not to acquire a plethora of crockery to be housed in one or, at most, two rooms.

Nurses must beware of running a system to rags—as when "Patricia" speaks of a piece of paper pinned on the wall with her stated requirements (a very sensible arrangement)—being previously disinfected! Her own hands must eventually place it there, so that the disinfection can only be partial. Avoid the mistake of piling up endless precautions, which with a case lasting from six to eight weeks will inevitably break down.

### Honourable Mention.

Six candidates win this—"Febris Rubra," "Enfield,' "Brum," "Lanoitan," "Central," and "Festina Lente.' "Febris Rubra" would have gained the first prize, but her paper is disqualified for printing by having marginal notes.

The practice of dividing answers, putting headlines and marginal notes has been frequently prohibited.

Faults in Some Papers.

Several nurses say they should put the end of the carbolised sheet outside the door in a footbath, so that by suction it should always remain wet. This plan will not answer, because it would leave the bottom of the door, where the draught is greatest entirely unprotected.

Question for November.

1. If requested by a doctor to strap a leg for the cure of an ulcer half way up the shin, how would you proceed? Describe your preparation of the limb—of the strapping—and of your application of the strapping to the leg.

2. In what manner should you remove the plaster when it required removing? The Examiner.

Rules.

The competition is open to all. Answers must not exceed 500 words, and must be written on one side of the paper only, without divisions, head lines, or marginal notes. The pseudonym, as well as the proper name and address, must be written on the *same* paper, and *not on a separate sheet.* Papers may be sent in for fifteen days only from the day of the publication of the question. All illustrations strictly prohibited. Failure to comply with these rules will disqualify the candidate for competition. Prizes will be awarded for the best two answers. Papers to be sent to "The Editor," with "Examination" written on the left-hand corner of the envelope.

In addition to two prizes honourable mention cards will be awarded to those who have sent in exceptionally good papers.

N.B.—The decision of the Examiner is final, and no correspondence on the subject can be entertained.

Any competitor having gained three prizes within the current year shall be disqualified from taking another until 12 months shall have expired since the first prize was gained.

### The Mistake of a Plaistow Probationer.

The account in last Saturday's *Daily News* of the inquest held by the West Ham coroner concerning the death of a patient at St. Mary's Children's Hospital, Plaistow, calls for some explanation. According to our contemporary, a little boy of five, who had been scalded, was taken by his mother to St. Mary's Hospital, where, treated as an out-patient, his wounds were dressed by a probationer who let him go away without seeing a doctor. He was brought again to the hospital the next day when he was seen by the hospital doctor. The following morning the child seemed very ill, and when he was once more taken to the hospital he was admitted, and died soon afterwards. The probationer who had in the first instance attended to the child had to give evidence at the inquest, and one of the coroner's questions was: "Is it your practice to dress wounds without the doctor seeing the patients?" To which the probationer replied, "the doctor was engaged at the time the child was brought in, on Sunday, but on Monday I sent the child to see the doctor."

The coroner said that the father of the child wanted to know if it was the custom at the hospital to dress wounds and allow the patients to leave before being seen by the doctor. To this the probationer answered that it was not the custom, but that as the wounds did not appear to be serious the sister thought that they might be dressed by a probationer. A juror said, "Don't you think that as there is always a great fright after scalds the child should have been seen by the doctor?" And the nurse returned: "Yes, I do, but these scalds were on the arms, and the shock is not so great then as it would be if they were on other parts of the body." The verdict was "Death from scalding," and the jury added that they thought it would be better for a certificated nurse to see the patients. The coroner said, "I think that a doctor should see the patients, but in this case a misunderstanding happened."

Our representative, who called at St. Mary's Hospital, Plaistow, has seen both the secretary and the matron, and was informed that, briefly, the facts are as follows: The child was brought to the hospital on a Sunday about midday. The probationer misunderstood the sister's order. She thought that the sister told her to dress the child's wounds, whereas the sister meant her to cover the wounds up until the doctor could see them. On Monday the child was seen by the doctor, who did not think it necessary to detain him as an in-patient. On Tuesday, when he came again, he was admitted and died shortly afterwards from septicæmia. The rule of the hospital is that no case, however trivial, shall go away without having been seen by the doctor; but most unfortunately, in this instance the probationer misunderstood the sister's order.

### Meeting of the Central Midwives Board.

A meeting of the Central Midwives Board was held at the offices, 6 Suffolk Street, on Thursday last week. There were present Dr. Champneys (Chairman), Mr. Ward Cousins, Miss R Paget, Mr. Parker Young, and Miss Wilson. Before proceeding to business the Board passed a vote of sincere sympathy with Mrs. J. Heywood Johnstone, moved from the chair and seconded by Mr. Parker Young.

The correspondence before the Board included several communications dealing with the personal character of certain midwives. It was agreed that it would be well to consider such matters *in camera*, to take the rest of the business on the agenda first, afterwards asking the Press to withdraw.

A letter was read from a county medical officer, notifying the suspension of a certified midwife, and asking the opinion of the Board as to whether midwives attending cases only with a doctor are bound to notify the local supervising authority of their intention to practise. The Board agreed that Section 10 of the Act referred to women practising as midwives, and not as monthly nurses, and that women certified as midwives, but not practising or holding themselves out as practising, cannot be compelled to notify.

Letters from institutions which the Board had been unable to recognise for the training of midwives, inquiring the reasons for such refusal, were then considered. The Board agreed that while it was impossible to give reasons, the secretary should forward in each case a marked copy of the rules giving the Board's requirements with regard to the number of cases to be conducted by each pupil midwife, from which the institutions would see that failure to comply in this respect was the cause of the Board's decision.

The financial statement was passed, and the applications for admission to the roll of 1,236 midwives were approved. This brings the total number upon the roll to 9,470, of which 2,953 hold the L.O.S. certificate and 5,652 are admitted under the *bonâ-fide* clause of the Act. The Portsmouth and Woolwich hospitals for soldiers' wives and children were approved for the training of midwives under Section C. of the rules.

The Board then considered the adjourned scheme of examinations, which was finally approved with a few unimportant alterations, the chief of these being the decision to hold the first examination in July instead of May 1905. The Board agreed that the approved scheme should be published in the medical papers in due course.

The following resolution was moved by Miss Paget, seconded by Miss Wilson, and passed:—

That the period of the approval of any certified midwife for the purpose of signing Forms III. and IV. under Rule C. I. (2) shall expire on March 31st following such approval, but may be renewed from time to time for the period of one year as often as the Board shall think fit, provided that no approval granted by the Board before March 31st, 1905, shall require renewal before March 31st, 1906.

It was agreed to order the issue of 500 copies of the "Register of Midwives," at 10s. 6d. a volume. The Board then agreed to hold an adjourned meeting for the consideration of the matters to be discussed *in camera*, and a number of applications for recognition as teachers under Rule C. I. (3) and as certified midwives for the purpose of signing Forms III. and IV. under Rule C. I. (2).

### Appointments.

[No charge is made for announcements under this head, and we are always glad to receive, and publish, appointments. The information, to insure accuracy, should be sent from the nurses themselves, and we cannot undertake to correct official announcements which may happen to be inaccurate. It is essential that in all cases the school of training should be given.]

Bermondsey Schools, Wickham Road, Shirley.—Miss Emily Read and Miss Maud Elizabeth Jane Wood have been appointed charge nurses. Miss Read was trained at the National Orthopædic Hospital, London, and has since been charge nurse in the parish of Bermondsey. Miss Wood was trained at the Royal Free Hospital, London, and has since been nurse at the Passmore Edwards District Cottage Hospital, Tilbury, and night superintendent at the Croydon Union Infirmary.

City Hospital (North), Liverpool.—Miss Lydia Watson has been appointed sister. She was trained at the Royal Infirmary, Liverpool, and has since been private nurse at Mildmay Park, London, charge nurse at Eastham Fever Hospital, and sister at Liscard Isolation Hospital.

Colwyn Bay Cottage Hospital.—Miss Kate Jones has been appointed nurse-matron. She was trained at the Liverpool Royal Infirmary, where she has since been ward sister. She has since acted as temporary night superintendent and temporary housekeeper at the same institution.

Grantham Hospital.—Miss Maud Williams has been appointed sister. She was trained at Chester General Infirmary, and has since been staff nurse at the Children's Hospital, Pendlebury, Manchester, and temporary out-patient sister at the Children's Hospital, Nottingham.

Hospital for Women, Soho, London.—Miss Alice Manley has been appointed staff nurse. She was trained at Addenbrooke's Hospital, Cambridge, and for midwifery at the East-end Mothers' Home, Commercial Road, London.

Morley House, Convalescent Home, St. Margaret's Bay, Devon.—Miss Florence Capes has been appointed superintendent matron. She was trained at St. George's Union Infirmary, Fulham, and has since been charge nurse at Sutton Schools; charge nurse at St. Olave's Infirmary; assistant matron and matron at St. Olave's; and matron at St. Michael's Orphanage, Woodside, Croydon.

North Devon Infirmary, Barnstaple.—Miss Ada Davies has been appointed sister. She was trained at the General and Eye hospital, Swansea.

Stroud Joint Hospital for Infectious Diseases.—Miss Margaret Mumford has been appointed matron. She was trained at the General Hospital, Birmingham, and has since been charge nurse at the South-Eastern Fever Hospital, London, and matron at the Borough Isolation Hospital, Ipswich.

### Everybody's Opinion.

### CLASS LEGISLATION.

Miss E. L. C. Eden writes from 19 St. James's Square, Bath:—In answer to "Inquirer's" letter in last week's issue, I beg to say that I know at least three nurses who have not had full training who are in favour of State registration. Surely a principle remains the same however it may affect one personally. The mere suggestion that this is not so strikes one as quaint.

### NURSING IN A COUNTRY INFIRMARY.

"Lady Superintendent" writes: Having read the letter which "M. E." writes concerning "Nursing in a Country Infirmary," I beg to state that after seven years' experience as superintendent of a home for invalid ladies I can fully corroborate her statements and can truthfully testify to its not being the least exaggerated, as we also have had to cope with the "serious difficulty" of securing suitable nurses, and should be only too glad if anyone could suggest a remedy.

### NURSES FOR THE MIDDLE CLASSES.

"Nurse M. O." writes:—Allow me to write a few lines after reading in the "Outlook" "Nurses for the Middle Classes." It is one of the most practically benevolent and broad-minded articles I have read for a long time. I daresay many nurses feel, as I do, that however true, sympathetic, and disinterested as regards pecuniary matters a nurse may be at the beginning of her career, the filth of the slums, or the thoughtless treatment accorded by the two and three-guinea payer, makes her sometimes feel after a ten years' service—when she has done all she could—that she was not wise to enter into a career where one has to draw so constantly on the capital of one's strength and endurance. Therefore, I think the scheme of forming homes for the middle classes, and giving daily visits, seems to me one of the grandest ideas, both as regards the people and the nurses, who after a life of devotion to their work, seem to be perfectly ignored after the age of thirty-five by most of the nursing homes, and seem not even desirable for chronic cases. I should be much obliged if you would put me into communication with those to whom I could apply for such a post, having thought the matter over for the last few years.

[The scheme to which the article referred is not at present sufficiently advanced for us to mention names. — Ed. Hospital.]

### "GOOD NURSING."

"An Old Charge-Nurse" writes: Perhaps the kindly-disposed "Night Superintendent," would like to try the way we were trained, to prepare abdominal sections. Of course, I speak only on the points which she takes up, and there are exceptions to all rules. Our general rule was to give the castor-oil two nights previous to the morning of operation (*i.e.* from 36–40 hours before). Usually the medicine did not disturb the patient until next morning. Should the result not be satisfactory an enema was given them. All the preparations for the section were done by the day-nurses, except the enema and douche on the morning of operation. The patient had a cup of beef tea, four hours before operation, and half an hour before 1 oz. of brandy without water. Collapse was very rare indeed, and deaths very few. In a case such as "Night Superintendent" cites, Mrs. D—— comes in on Tuesday, she would get a dose that night, though not usually castor-oil. If, on the Wednesday, the surgeon said he would operate on Thursday, then the first night's dose would do, if not sufficient another might be given, but *at once*, or an enema of oil during the afternoon. Might I suggest, if the surgeon was in the habit of giving orders, to prepare for operation the day following, that the house-surgeon or sister in charge give the required dose on the patient's arrival? I was privileged to do so when in charge.

### Echoes from the Outside World.

### Movements of Royalty.

The King, after spending several hours on Saturday shooting over the Chippenham Park Estate, returned from Cambridgeshire to London in the evening, arriving at St. Pancras Station a little after seven. The King sent for the Prime Minister to Buckingham Palace on Monday, before the meeting of the Cabinet, and after the Council Lord Lansdowne had an audience of his Majesty, lasting a considerable time. During the day Mr. Soloman, A.R.A., submitted for the King's inspection a picture he has painted of her late Majesty Queen Victoria. The Queen, who is at Sandringham, has been busily engaged in superintending the interior arrangements of the private apartments which have recently been completed, after undergoing extensive alterations and redecorations. The Prince of Wales, who has been shooting in his private preserves which adjoin the Sandringham estate, is the guest of Lord and Lady Iveagh at Elvedon this week; and the Princess of Wales has returned to Marlborough House. Princess Christian and Princess Victoria of Schleswig-Holstein arrived home on Saturday.

### Great Britain and Russia.

The crisis in the relations between England and Russia arising from the outrage on the Hull fishing fleet was ended on Friday, the Prime Minister having had an interview with the Queen after the Cabinet Council on that day made a full statement which he prefaced by expressing his belief that "so far as he was able to foresee the future, the lamentable and deplorable tragedy of the previous Friday would not end in one of those great national struggles which, though they might now and again be necessary, always left a deplorable mark behind them, and retarded the progress of humanity and civilisation." Mr. Balfour described the report of the Russian Admiral as containing much romance, but two versions of the incident having been put before them, the matter was to be made the subject of an international and impartial inquiry. The truth would, he hoped, be made manifest when the inquiry which the Tsar welcomed takes place The Russian Ambassador, he continued, authorised him to announce that his Government and the Tsar, on hearing of the North Sea incident, at once expressed their profound regret. They promised liberal compensation to the sufferers, had ordered the detention at Vigo of the section of the Russian fleet responsible for the disaster, and pledged themselves that any person found guilty by the responsible tribunal would be adequately punished. Mr. Balfour added that the Russian Government had shown an enlightened desire that truth and justice in this matter should prevail. Count Lamsdorff on the same day telegraphed to the Russian Ambassador in London that the Tsar in his desire to bring everything that occurred in the North Sea into as clear a light as possible, considered it beneficial to submit the affair to an International Commission of the Inquiry on the basis of The Hague Convention, and the Ambassador was instructed to prepare this means for the settlement of the question to the British Government. Count Benckendorff replied late in the day that the British Government accepted the proposal. Russia, it is stated, is prepared to allow £100,000 as an indemnity to the British fishermen.

### The War in the Far East.

Marshal Oyama reports that on Thursday last week a detachment of the Japanese Right Army attacked the Russians at Wai-tau-shan, and, after meeting with stubborn resistance, occupied the position, capturing two machine-guns. On the following day the Russians cannonaded Wai-tau shan till afternoon, and then dispersed. On Friday night the Russians made a counter-attack on Wai-tau-shan, but were at once repulsed. On Saturday a force from the Centre Army seized and burned a hamlet midway between the lines of the two armies which the Russians had constantly used as a base of attack against the Japanese outposts.—According to the Russian General Staff, the Russian losses in the Battle of the Sha-ho were 800 officers and 45,000 men killed, wounded and missing.—Japanese financiers estimate the war requirements for the coming year at £77,000,000, of which they expect to obtain £22,000,000 from surplus of ordinary revenue and war taxes, and the remainder from loans.—General Kuropatkin, in a telegram of Sunday, reports a movement eastward of the Japanese forces, and says that the Japanese are also reported to be receiving reinforcements from the south and from the direction of Feng-hwan-chenn. From these reports it is concluded that the Japanese are completing concentration of their forces in order to assume the offensive.

### Resignation of the Bishop of Gloucester.

It is officially announced that the Bishop of Gloucester will resign his see on Lady Day next. Dr. Ellicott, who is 85 years old, was, 56 years ago, a professor at King's College, London. In 1861 he was made Dean of Exeter, and for over 41 years he has been Bishop of Gloucester. He acted as Chairman of the Committee for the Revision of the New Testament, has been a prominent figure in both Houses of Convocation, and though frail in appearance, he has survived the effects of a terrible accident in his early life and been a very active man. When Archbishop Langley died his name was submitted by Mr. Disraeli with that of Bishop Tait for the Primacy, Queen Victoria choosing the latter.

### Mr. Abbey's Coronation Picture.

By permission of the King, the picture of the Coronation, which has been painted by Mr. Edwin Abbey is now exhibited at the Hanover Gallery in New Bond Street. The canvas measures 15 feet by nine feet, and the scene represents the moment in Westminster Abbey when Archbishop Temple aged yet dark-haired, is about to place the crown on King Edward's head. His Majesty, attired in the gorgeous robes, sits in the centre, and to the left is Queen Alexandra, in cloth of silver, with the Duchess of Buccleuch, mistress of the robes. Above in the Royal tribune are the King's daughter-in-law and daughters, with their children, and the other princesses, while behind the King's chair are the Prince of Wales and Duke of Connaught. The great officers of State, the peers and peeresses of the highest rank, fill a considerable portion of the picture, and, in fact, no detail is omitted that could possibly be included. The interior of the Abbey is very faithfully depicted, and altogether the work is singularly effective and beautiful, reflecting the greatest credit on the artist. After it has been exhibited in London and other British cities, in some of the colonies, and in the United States of America, it will find a permanent home at Buckingham Palace or Windsor.

### Death of Mr. Dan Leno.

A great popular favourite died on Monday in the person of Mr. Dan Leno, the comedian and comic singer. His real name was Gavain, and his parents were music-hall artists, who were known as Mr. and Mrs. Johnny Wilde. He first appeared on a music-hall stage when he was only three years old, and continued to sing at music-halls more or less till he went to Drury Lane for pantomime, and made a great success. His health first broke down in 1902, but a few months previously he had the honour of being commanded to give his performance at Sandringham before the King and members of the Royal Family.

### Notes and Queries.

**FOR REGULATIONS SEE PAGE 12.**

*Nitrate of Silver.*

(36) Will you please tell me, (1) why does nitrate of silver turn ordinary water milky, and not sterilised water. (2) Does not water have to go through a special process more than mere boiling to make it sterilised?—*M. C.*

Ordinary (tap) water contains salts in solution which produce insoluble precipitates with nitrate of silver. Water which is sterilised is only boiled and cooled without access of air, or with access of filtered air only. Consequently sterilised water will also yield precipitates with nitrate of silver. Distilled water, which contains no salts in solution, gives no precipitate with nitrate of silver.

*Adoption of Child.*

(37) I want to adopt a child of good family, from one month or older. Will you kindly let me know the best way to get one either by advertisement or otherwise?—*A. S.*

If you cannot hear of one privately advertise.

*Defective Eyesight.*

(38) Will you kindly tell me whether a slight defect of eyesight (astigmatism), which makes the constant use of glasses advisable, is of necessity a barrier to my being trained as a nurse?—*F. E.*

Not necessarily. Some hospitals are more particular in this respect than others.

*Home for Tuberculosis Patient.*

(39) I should be obliged if you could tell me of a home on or near the South Coast for a young man suffering from tuberculosis in the knee, and therefore requiring slight nursing in assisting to dress, etc.?—*R. E. B.*

See the list of Sanatoria, published by the National Association for the Prevention of Tuberculosis, obtainable from the Scientific Press, price 7d., with postage.

*Duties.*

(40) Will you kindly tell me what are the duties of a matron at the opening ceremony of a new hospital?—*A. McR.*

It is impossible to enumerate them. They would depend upon the hospital, by whom the new buildings were opened, and circumstances generally.

*Training.*

(41) I should be much obliged if you would kindly give me some information on the following points:—(1) Is there any special reason why the training given at a London hospital is to be preferred to that of a provincial one? (2) What constitutes "a recognised training school?" (3) Is it advisable to gain a certificate in massage and a diploma from the L.O.S. in addition to a three years' training in a general hospital?—*Scolopax.*

(1) It is a question of professional *status* really, London ranking before the provinces. (2) A hospital containing not less than 100 beds, and having resident medical men. (3) It is generally advisable to get every additional certificate possible, so as to be fully equipped.

*Massage Certificate and Catheter.*

(42) 1. Would it be better to go through a course of massage training at the National Hospital and get their certificate, or to qualify by private tuition and get that of the Society of Masseurs? 2. What are the special different points to be observed in each case in passing a catheter after (*a*) the operation for ruptured perineum; (*b*) abdominal operation?—*Nil Desperandum.*

1. A matter of preference. 2. (*A*) A case of ruptured perineum is kept lying down, with a bandage round the legs above the knees. Therefore a catheter must be passed with the patient lying on her side. (*B*) In abdominal operations it is usual to pass a catheter more frequently, say once in every four, instead of six, hours, that the bladder may never be sufficiently distended to cause any tension on the stitches.

**Handbooks for Nurses.**

"A Handbook for Nurses." By Dr. J. K. Watson. 5s. net; 5s. 4d. post free.

"A Complete Handbook of Midwifery." By Dr. J. K. Watson. 6s. net; 6s. 4d. post free.

"The Nurses' Pronouncing Dictionary." 2s. post free.

"Elementary Anatomy and Surgery for Nurses." Price 2s. 6d.

How to Become a Nurse: "The Nursing Profession." 2s. 4d. post free.

The above may be obtained of all booksellers or of The Scientific Press, Limited, 28 & 29 Southampton Street, Strand, London, W.C.

### For Reading to the Sick.

### SUPPLICATION.

We have not known Thee as we ought,
Nor learn'd Thy wisdom, grace, and power;
The things of earth have fill'd our thought,
And trifles of the passing hour.
Lord, give us light Thy truth to see,
And make us wise in knowing Thee.

We have not loved Thee as we ought,
Nor cared that we are loved by Thee;
Thy presence we have coldly sought,
And feebly long'd Thy Face to see.
Lord, give a pure and loving heart
To feel and own the love Thou art.

When shall we know Thee as we ought,
And fear, and love, and serve aright!
When shall we out of trial brought
Be perfect in the land of light!
Lord, may we day by day prepare
To see Thy Face and serve Thee there.

*T. B. Pollock.*

### PRAYER.

Lord, give us grace, we beseech Thee, to hear and obey Thy voice, which saith to every one of us, "This is the way, walk ye in it." Nevertheless, let us not hear it behind us saying, "This is the way"; but rather before us, saying "Follow me." When Thou puttest us forth, go before us; when the way is too great for us, carry us; in the darkness of death comfort us; in the day of resurrection satisfy us.—*Christina Rossetti.*

"The whole earth is full of the character of the Lord." Christ is the Light of the world, and much of His Light is reflected from things in the world—even from clouds. Sunlight is stored in every leaf, from leaf through coal, and it comforts us thence when days are dark and we cannot see the sun. Christ shines through men, through books, through history, through nature, music, art. Look for Him there. "Every day one should either look at a beautiful picture, or hear beautiful music, or read a beautiful poem."

In the Galerie des Beaux Arts in Paris there stands a famous statue. It was the last work of a great genius, who, like many a genius, was very poor and lived in a garret, which served as studio and sleeping-room alike. When the statue was all but finished, one midnight a sudden frost fell upon Paris. The sculptor lay awake in the fireless room and thought of the still moist clay, thought how the water would freeze in the pores and destroy in an hour the dream of his life. So the old man rose from his couch and heaped the bed-clothes reverently round his work. In the morning when the neighbours entered the room the sculptor was dead. But the statue lived.

The Image of Christ that is forming within us—that is life's one charge. Let every project stand aside for that. "Till Christ be found, no man's work is finished, no religion crowned, no life has fulfilled its end." Is the infinite task begun? When, now, are we to be different? Time cannot change men. Death cannot change men. Christ can. Wherefore *Put on Christ.—From "The Changed Life."*

